# Virologic failure and mortality in older ART initiators in a multisite Latin American and Caribbean Cohort

**DOI:** 10.1002/jia2.25088

**Published:** 2018-03-22

**Authors:** Gabriela Carriquiry, Mark J Giganti, Jessica L Castilho, Karu Jayathilake, Pedro Cahn, Beatriz Grinsztejn, Claudia Cortes, Jean W Pape, Denis Padgett, Juan Sierra‐Madero, Catherine C McGowan, Bryan E Shepherd, Eduardo Gotuzzo

**Affiliations:** ^1^ Instituto de Medicina Tropical Alexander von Humboldt Lima Peru; ^2^ Vanderbilt University Nashville TN USA; ^3^ Fundación Huésped Buenos Aires Argentina; ^4^ Instituto Nacional de Infectologia Evandro Chagas‐Fundação Oswaldo Cruz Rio de Janeiro Brazil; ^5^ Fundación Arriarán Santiago Chile; ^6^ Le Groupe Haïtien d'Etude du Sarcome de Kaposi et des Infections Opportunistes Port‐au‐Prince Haiti and Weill Cornell Medical College New York NY USA; ^7^ Instituto Hondureño de Seguridad Social and Hospital Escuela Universitario Tegucigalpa Honduras; ^8^ Instituto Nacional de Ciencias Médicas y Nutrición Salvador Zubirán Mexico City Mexico

**Keywords:** HIV, Ageing, Over 50, Latin America, Caribbean, ART outcomes, Virologic failure

## Abstract

**Introduction:**

The “greying” of the HIV epidemic necessitates a better understanding of the healthcare needs of older HIV‐positive adults. As these individuals age, it is unclear whether comorbidities and their associated therapies or the ageing process itself alter the response to antiretroviral therapy (ART). In this study, HIV treatment outcomes and corresponding risk factors were compared between older ART initiators and those who were younger using data from the Caribbean, Central and South America Network for HIV Epidemiology (CCASAnet).

**Methods:**

HIV‐positive adults (≥18 years) initiating ART at nine sites in Argentina, Brazil, Chile, Haiti, Honduras, Mexico and Peru were included. Patients were classified as older (≥50 years) or younger (<50 years) based on age at ART initiation. ART effectiveness was measured using three outcomes: death, virologic failure and ART treatment modification. Cox regression models for each outcome compared risk between older and younger patients, adjusting for other covariates.

**Results:**

Among 26,311 patients initiating ART between 1996 and 2016, 3389 (13%) were ≥50 years. The majority of patients in both ≥50 and <50 age groups received a non‐nucleoside reverse transcriptase inhibitor‐based regimen (89% vs. 87%), did not have AIDS at baseline (63% vs. 62%), and were male (59% vs. 58%). Older patients had a higher risk of death (adjusted hazard ratio (aHR) 1.64; 95% confidence intervals (CI): 1.48 to 1.83) and a lower risk of virologic failure (aHR: 0.73; 95% CI: 0.63 to 0.84). There was no difference in risk of ART modification (aHR: 1.00; 95% CI: 0.94 to 1.06). Risk factors for death, virologic failure and treatment modification were similar for each group.

**Conclusions:**

Older age at ART initiation was associated with increased mortality and decreased risk of virologic failure in our cohort of more than 26,000 ART initiators in Latin America and the Caribbean. To the best of our knowledge this is the first study from the region to evaluate ART outcomes in this growing and important population. Given the complexity of issues related to ageing with HIV, a greater understanding is needed in order to properly respond to this shifting epidemic.

## 1 Introduction

Worldwide, HIV‐positive populations are ageing. In 2013, an estimated 13% of the global adult population living with HIV was 50 years and older (defined as “older adults” in HIV literature); increasing proportions of older HIV‐positive adults have been observed in all regions, ranging from 10% in low‐ and middle‐income countries to 30% in high‐income countries. By 2012, the estimated percentage of adults living with HIV who were over the age of 50 was 15% in Latin America and 13% in the Caribbean 1.

In 2013, people aged 50 and over accounted for 18% of new HIV diagnoses in the United States (U.S.) 2. In addition to sharing many of the same HIV risk factors as younger people, older people face several unique issues. Women no longer concerned about pregnancy may be less likely to practice safe sex 3, 4. Erectile dysfunction medications may increase the number of sexual interactions for older men 5. Older people may have an inaccurate perception of the risk for HIV infection in their peer group and less frequent discussions with medical providers regarding sexual practices and drug use 6, 7, 8. Finally, age‐related changes in the reproductive tract and immune system may enhance susceptibility to HIV acquisition 1, 9.

Older HIV populations present unique challenges at all stages of the care continuum‐ timely diagnosis, linkage to care, and for those retained on antiretroviral therapy (ART), frequent co‐morbidities and polypharmacy. Studies from the U.S. and Canada have demonstrated that older adults often present with lower CD4 counts and are more likely to have an AIDS‐defining condition at the time of HIV diagnosis compared to their younger counterparts 10. Once started on ART, older adults have less CD4 recovery despite similar rates of virologic suppression and higher rates of ART adherence 11, 12. Recent mortality studies have shown that ART initiation at higher CD4 counts may be particularly important for older adults starting therapy 13. In addition, as HIV‐positive adults age they have high accumulation of co‐morbid conditions, including renal and liver disease, and co‐medication use, that may affect their response to ART 14, 15.

As ART coverage and access to HIV care continues to expand, an increased awareness of the clinical needs of older persons living with HIV is required in the context of different regional settings. In the last few years, ART programmes have achieved coverage of 47% and 44% of all HIV‐positive adults (15 years and older) in Latin America and the Caribbean respectively 16. In many low‐ and middle‐income countries, epidemiologic shifts from infectious to non‐communicable disease (including cardiovascular disease, renal disease and cancers) are occurring concurrently among persons living with HIV and may particularly affect ART outcomes of older HIV‐infected adults 17, 18, 19.

In this study, we analysed data from the Caribbean, Central and South America Network (CCASAnet) cohort to describe risk factors for disparities in outcomes (including death, ART modification and virologic failure) following ART initiation in older relative to younger adults living with HIV in Latin America and the Caribbean (LAC).

## 2 Methods

### 2.1 Cohort description

This retrospective observational cohort study included HIV‐positive adults (≥18 years) who initiated ART between 1996 and 2016 at nine CCASAnet sites: Hospital Fernández and Centro Médico Huésped in Buenos Aires, Argentina (HF/CMH‐Argentina) (data only available up until 2014); Instituto Nacional de Infectologia Evandro Chagas in Rio de Janeiro, Brazil (INI‐Brazil); Fundación Arriarán in Santiago, Chile (FA‐Chile); Le Groupe Haïtien d'Etude du Sarcome de Kaposi et des Infections Opportunistes in Port‐au‐Prince, Haiti (GHESKIO‐Haiti); Instituto Hondureño de Seguridad Social and Hospital Escuela Universitario in Tegucigalpa, Honduras (IHSS/HE‐Honduras); El Instituto Nacional de Ciencias Médicas y Nutrición Salvador Zubirán in Mexico City, Mexico (INNSZ‐Mexico); and Instituto de Medicina Tropical Alexander von Humboldt in Lima, Peru (IMTAvH‐Peru). The CCASAnet cohort has been previously described 20.

### 2.2 Ethics and data management

Institutional ethics review boards from each participating site and Vanderbilt University reviewed and approved the project. Informed consent process was done at IMTAvH‐Peru and waived at all the other sites.

Clinical, laboratory and demographic data were collected at each centre, de‐identified and sent to the CCASAnet Data Coordinating Center at Vanderbilt University (VDCC), Nashville, TN, U.S. for data cleaning, processing and merging. The VDCC checked data for internal consistency and performed on‐site data audits to verify accuracy of data received with that contained in the medical record 21.

### 2.3 Study definitions and outcomes

The baseline time point for this study (“ART initiation”) was defined as the earliest time of starting ART at the CCASAnet clinical site. ART was defined as a combination of at least three antiretroviral drugs, where at least two drugs came from different classes or at least three drugs were nucleoside/nucleotide reverse transcriptase inhibitors. Patients were classified as older (≥50 years) or younger (<50 years) based upon their age at baseline. The designation of “older” HIV‐positive patients as ≥50 years old is based on the definition used by the United States Centers for Disease Control 22.

The following patient characteristics at baseline were also of interest due to their potential association with age at ART initiation and HIV outcomes: sex, presence of AIDS‐defining illness, probable route of HIV transmission, ART regimen, time from HIV diagnosis to ART initiation, calendar year, plasma HIV‐1 viral load (VL) and CD4 count. Baseline VL and CD4 count were defined for each patient as the measurement closest to ART initiation, no more than 180 days before or seven days after.

ART effectiveness was measured using three outcomes: death, virologic failure and ART treatment modification. The time from ART initiation to event was calculated separately for each outcome. If a patient did not have an event, they were censored at the first of 31 December 2016 or the date of their last clinical, pharmacy, or laboratory visit. A patient was defined as lost to follow‐up (LTFU) if they had zero visits within the last 365 days that data was available for their respective cohort.

Determination of virologic failure was based on whether the patient experienced at least one of the following conditions: (1) no VL measurements below 400 copies/ml after six months of therapy, (2) a VL measurement above 1000 copies/ml after at least one VL measurement below 400 copies/ml, (3) or two consecutive values greater than 400 copies/ml after at least one VL measurement below 400 copies/ml. The threshold for classifying a measurement as undetectable was set at 400 copies/ml to account for changing detection limits of assays used at many of the sites over time. The second condition was added because it corresponds with clinical practices at some sites in the region 23. As VLs were not routinely performed at GHESKIO‐Haiti, all patients from this site were excluded from the virologic failure analysis. Patients from other sites were also excluded from the virologic failure analysis if they did not have a VL measurement after ART initiation. When the gap between measurements was greater than one year, patients were censored at the last VL measurement prior to the gap. Treatment modification was defined as any drug dispensation change, including both single and multiple drug substitutions.

### 2.4 Statistical analysis

Patients were excluded from the analysis cohort if they had ART exposure prior to ART initiation or an undetectable VL measurement at baseline. Baseline demographic, clinical, and laboratory characteristics of older and younger patients were compared using Wilcoxon Rank Sum and Chi‐square tests for continuous and categorical variables respectively. The proportion of ART initiators who were over 50 years over time was estimated for each site using a loess smoother.

Inverse‐probability weighted estimates of the median and interquartile range (IQR) CD4 count were calculated at 6, 12 and 24 months for older and younger patients with a corresponding lab measurement at baseline. CD4 count levels at each time point were determined by available laboratory values closest to the defined time ±90 days. Patients who died prior to each time point were assigned to have the worst rank measurements (i.e. the lowest CD4 measurement) 24, 25. The probability of a missing value was modelled separately for each laboratory measurement at each time point using logistic regression models that included covariates for site, age, sex, presence of AIDS‐defining illness, year of ART initiation, ART regimen, probable route of HIV transmission and the corresponding baseline laboratory measurement. To account for differences between age groups, the probability of being in the older patient group given baseline values was also estimated using logistic regression. Weights for each observation were calculated by multiplying the inverse probability of a non‐missing lab value by the inverse probability of being in the observed age group; final weights were winsorized at the 99th percentile. *p*‐values comparing age groups at each time point were calculated using weighted Wilcoxon and Chi‐square tests using the “survey” package in R 26.

Kaplan–Meier estimates and 95% confidence intervals (CI) for the probability of death over time were calculated for each age group. The cumulative incidence and corresponding 95% CI of treatment modification and virologic failure, accounting for mortality as a competing risk, were estimated for each age group. Treatment modification cumulative incidence estimates were also calculated for specific periods of ART initiation (1996 to 2001, 2002 to 2006, 2007 to 2011, 2012 to 2016).

Separate multivariable Cox regression models were fit to compare the cause‐specific hazard of an event between older versus younger HIV‐infected patients. Covariates for adjustment were chosen a priori as potential confounders based on their clinical relevance and availability, and included sex, clinical AIDS at baseline, CD4 count, ART initiation year, ART regimen class, intravenous drug use as HIV transmission risk, time from HIV diagnosis to ART initiation. To account for differences in the underlying hazard function between sites, adjusted Cox models were also stratified by cohort 27. As a sensitivity analysis, separate models were fit using data from each age group and period of ART initiation. Restricted cubic splines with four knots were used to relax linearity assumptions for continuous covariates. To assess the proportional hazards assumption, we used the “cox.zph” function from the “survival” package in R 28 to estimate the correlation coefficient between transformed survival time and the scaled Schoenfeld residuals corresponding to the age group covariate.

Age‐specific mortality rates (per 100,000 person‐years) were calculated for the entire cohort as well as for each individual country. The age groups (20 to 34, 35 to 44, 45 to 54, 55 to 64, 65 to 74, 75+) were chosen to correspond with the age‐specific mortality rates (per 100,000 individuals) reported by the Pan‐American Health Organization (PAHO) 29. We tabulated the length of time that each patient was in a given age interval between their time of treatment initiation and end of follow‐up as well as the number of patients that died for each age‐interval. CI was calculated using a bootstrapping procedure.

To account for missing covariate values, a multiple imputation procedure with 10 imputation replications was implemented using the “mi” package in R 30. This package uses a chained equation approach to multiply impute missing values. Imputations were generated using both baseline covariates (sex, clinical AIDS at baseline, square‐root transformed nadir CD4 count, calendar year of ART initiation, indicator variables for each ART regimen class at treatment initiation, intravenous drug use as HIV transmission risk, time from HIV diagnosis to ART initiation) and the respective outcomes (event indicator, log‐transformed time of event or censoring, and interaction of the event indicator and time). For the virologic failure analysis, a separate imputation was performed among just the cohort of patients that met inclusion criteria. This multiple imputation procedure assumes that data were missing at random.

All reported *p*‐values are two‐sided. Analyses were performed using R Statistical Software (http://www.R-project.org); analysis scripts are available at http://biostat.mc.vanderbilt.edu/ArchivedAnalyses.

## 3 Results

### 3.1 Patient characteristics

A total of 26,311 adult ART initiators were included in this study; 3389 (13%) were ≥50 years. The percentage of patients ≥50 years was similar across cohorts: 9% in INNSZ‐Mexico, 10% in IHSS/HE‐Honduras, 10% in IMTAvH‐Peru, 11% in INI‐Brazil, 11% in FA‐Chile, 12% in HF/CMH‐Argentina, and 15% in GHESKIO‐Haiti.

An additional 9807 adults were enrolled in the cohorts, but were excluded from analysis; 4722 patients (4376 < 50 years old and 346 ≥ 50 years old) never initiated ART and 5085 (4814 vs. 271) were not ART‐naïve.

Baseline patient characteristics are shown in Table 1. Patients were predominantly male (59% of older patients and 58% of younger patients). The median age at ART initiation was 55 years (IQR: 52 to 59 years) for older patients and 34 years (IQR: 28 to 40 years) for younger ones. The proportion of patients with clinical AIDS prior to starting ART was 18% for older patients and 19% for younger patients. Older and younger patients had similar median CD4 count nadir (184 vs. 182 cells/μl) and baseline values (187 vs. 186 cells/μl). Older and younger patients also had similar VL (median log_10_ viral load of 5.0 for both groups). Baseline VL was missing for 70% and 64% of older and younger patients respectively, largely from the site in Haiti where VL was not routinely measured; among patients at other sites, baseline VL was missing for 30% and 31% of older and younger patients respectively. The median year of ART initiation was 2010 for both age groups. The majority of patients (89% vs. 87%) started a regimen containing a non‐nucleoside reverse transcriptase inhibitor (NNRTI) and time from diagnosis to ART start was 105 (27 to 750) days in older patients and 153 (35 to 873) days for younger patients (*p* < 0.001).

**Table 1:** Age‐stratified characteristics of patients who initiated antiretroviral therapy across nine Caribbean, Central and South America sites (n = 26,311)

	Over 50 (n = 3389)	Under 50 (n = 22,922)	*p*‐value
Age, years	55 (52 to 59)	34 (28 to 40)	
Sex			0.248
Male	2014 (59%)	13,378 (58%)	
Probable route of transmission			<0.001
Heterosexual	970 (29%)	5581 (24%)	
MSM	400 (12%)	5452 (24%)	
IDU	4 (0%)	198 (1%)	
Other	21 (1%)	75 (0%)	
Unknown	1995 (59%)	11,616 (51%)	
Clinical stage, baseline			0.179
AIDS	617 (18%)	4424 (19%)	
Not AIDS	2124 (63%)	14,244 (62%)	
Missing	648 (19%)	4254 (19%)	
Nadir CD4 count, cells/μl	184 (75 to 298)	182 (71 to 295)	0.157
Missing	422 (12%)	2663 (12%)	0.167
Baseline CD4 count, cells/μl	187 (78 to 304)	186 (73 to 303)	0.207
Missing	559 (16%)	3547 (15%)	0.133
Baseline viral load (log_10_)	5.0 (4.4 to to 5.5)	5.0 (4.4 to 5.4)	0.074
Missing	2385 (70%)	14,692 (64%)	<0.001
Baseline ART class			0.003
NNRTI	3008 (89%)	19,861 (87%)	
Boosted PI	275 (8%)	2244 (10%)	
Other	106 (3%)	817 (4%)	
ART initiation year	2010 (2007 to 2013)	2010 (2006 to 2013)	<0.001
Time from HIV diagnosis to ART initiation, days	105 (27 to 750)	153 (35 to 873)	<0.001
Missing	44 (1%)	255 (1%)	0.387

ART, antiretroviral therapy; IDU, injection drug use; MSM, men who have sex with men; NNRTI, non‐nucleoside reverse transcriptase inhibitor, PI, protease inhibitor.

**Figure 1:**
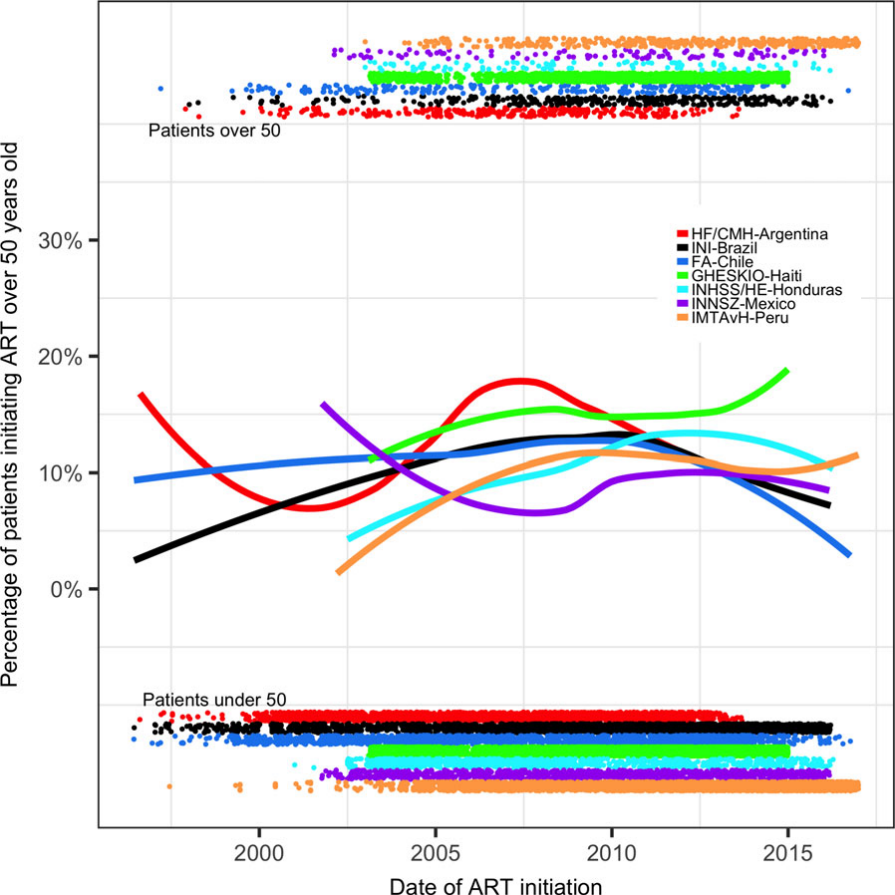
Estimated proportion of ART initiators older than 50 years during the study period. Points at top and bottom of the figure correspond to the date of ART initiation for individual patients over 50 years and under 50 years respectively. ART, antiretroviral therapy.

The estimated proportion of ART initiators who were over 50 years varied by site and date of ART initiation (Figure 1). The relative proportion of older patients increased over time at GHESKIO‐Haiti, IHSS/HE‐Honduras, and IMTAvH‐Peru. Proportions fluctuated at HF/CMH‐Argentina, INI‐Brazil, and FA‐Chile, but remained mostly unchanged over time. Only one site, INNSZ‐Mexico, had an estimated decrease in the relative proportion of older patients initiating ART over time.

Inverse probability weighted estimates for the median CD4 count at 6, 12 and 24 months since ART initiation are shown in Table 2. CD4 counts were similar between age groups at baseline after re‐weighting for baseline covariates, but CD4 recovery was slightly higher among younger patients. CD4 counts were statistically lower in older patients after six (median 287 vs. 302; *p* = 0.04), 12 (318 vs. 353; *p* < 0.001) and 24 months (321 vs. 374; *p* < 0.001).

**Table 2:** Changes in CD4 count over time by age group

	Over 50 (n = 2830)	Under 50 (n = 19,375)	*p*‐value
CD4 count, cells/μl			
Baseline	184 (71 to 299)	186 (74 to 303)	0.505
Six months^a^	287 (162 to 440)	302 (176 to 451)	0.044
12 months^a^	318 (181 to 485)	353 (218 to 524)	<0.001
24 months^a^	321 (131 to 496)	374 (199 to 550)	<0.001

ART, antiretroviral therapy.

a Inverse probability weighting was used to account for missing lab measurements.

**Figure 2:**
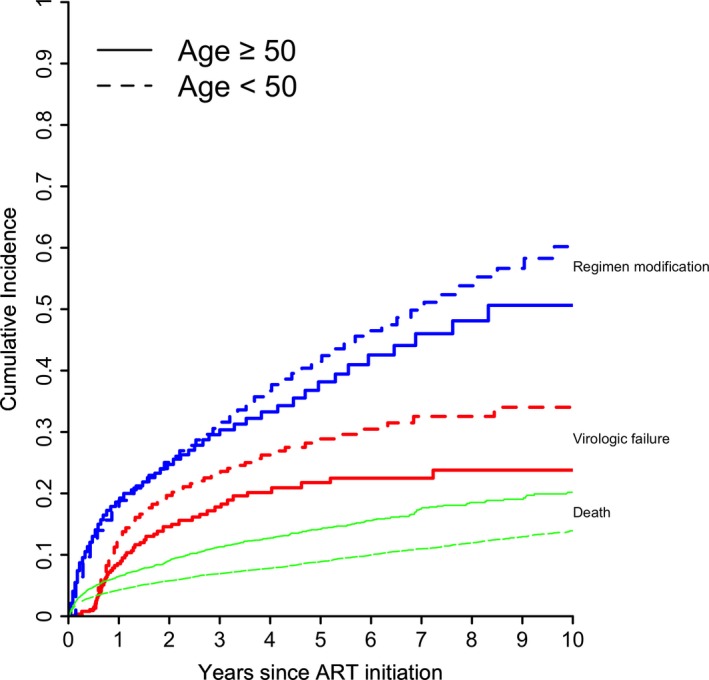
Crude cumulative incidence of mortality, regimen modification and virologic failure.

Cumulative incidence curves for mortality, ART modification and virologic failure are shown in Figure 2. Incidence of mortality was higher in older patients, while incidences of treatment modification and virologic failure were higher in younger patients. While the rate of ART modification has decreased over time, incidence rates for treatment modification remained slightly higher in younger patients across time periods (Figure S1).

Of the 26,311 patients that initiated treatment, 2383 (9%) died and an additional 9380 (36%) were LTFU during the study period. Overall mortality rates increased by age category, ranging from 1449 (95% CI: 1082 to 1833) per 100,000 person‐years for patients 20 to 24 years to 6201 (95% CI: 3507 to 9595) for patients 75 and older (Table S1). While mortality rates differed by country, trends according to age were similar across all sites. (Table S2).

In adjusted Cox proportional hazard models, older patients had a higher risk of death (adjusted hazard ratio (aHR) 1.64; 95% CI: 1.48 to 1.83) and a lower risk of virologic failure (aHR: 0.73; 95% CI: 0.63 to 0.84), after multiply imputing missing covariate values and adjusting for sex, presence of AIDS‐defining illness, intravenous drug use, ART regimen, time from HIV diagnosis to ART initiation, ART initiation year, and nadir CD4 count. There was no significant difference in the hazard of ART modification by age (aHR: 1.00; 95% CI: 0.94 to 1.06), although the data suggested that the association between age and ART modification changed over time (i.e. the proportional hazards assumption was violated). As a sensitivity analysis, we examined associations during the first three years after staring ART and then among those still remaining event free three years post‐ART initiation. Older patients had a non‐significant higher risk of ART modification during the first three years (aHR: 1.07; 0.99 to 1.15), but a lower risk after three years (aHR: 0.82; 95% CI: 0.72 to 0.93). The hazards of death and virologic failure were similar for both time periods (Table 3).

**Table 3:** Adjusted hazard ratios and corresponding 95% confidence intervals comparing older patients (≥50 years) versus a reference group of younger patients (<50 years) for death, ART modification, and virologic failure outcomes using different periods of follow‐up data

	Entire follow‐up period	During first three years of follow‐up	After first three years of follow‐up
Outcome			
Death	1.64 (1.48 to 1.83)	1.70 (1.50 to 1.92)	1.50 (1.22 to 1.84)
ART modification	1.00 (0.94 to 1.06)	1.07 (0.99 to 1.15)	0.82 (0.72 to 0.93)
Virologic failure	0.73 (0.63 to 0.84)	0.74 (0.63 to 0.86)	0.68 (0.47 to 0.99)

ART, antiretroviral therapy.

As shown in Table S3, clinical AIDS and a lower CD4 at baseline were predictive of an increased risk of death. Female sex, AIDS, lower CD4, earlier year of ART initiation, and starting a regimen that did not include NNRTI were predictive of ART modification (Table S3). Similarly, female sex, lower CD4, earlier year of ART initiation, and starting a non‐NNRTI regimen were associated with an increased risk of virologic failure. Age remained a strong predictor of mortality, even within age strata. (Tables S4 and S5). Among patients ≥50 years, the hazard of mortality was 48% higher for patients starting ART at age 60 years than at age 50. Among patients <50 years, the hazard of death was 24% lower for patients starting ART at age 40 compared to a similar patient starting just before their 50th birthday. When analyses were performed separately among adults ≥50 and <50 years, lower CD4 count was still associated with death and treatment modification, AIDS was still predictive of death, and PI‐based regimens remained a risk factor for treatment modification. Female sex was still predictive of both treatment modification (aHR: 1.30; 95% CI: 1.23 to 1.37) and virologic failure (aHR: 1.33; 95% CI: 1.20 to 1.47) in younger patients; however, associations were no longer significant among adults ≥50 years. Similarly, PI‐based regimens and CD4 were predictive of virologic failure and AIDS was predictive of treatment modification only among younger patients.

Four variables (intravenous drug use as HIV transmission risk, presence of AIDS‐defining illness, baseline CD4 count, and time from HIV diagnosis to ART initiation) had missing values for some patients and were imputed for analyses. Summary statistics comparing imputed values from a single replication with the observed values for the four incomplete variables are reported in Table S6. Results from an analysis that only included patients with complete data (n = 9304; Table S7) were similar for mortality (n = 9304; aHR for older vs. younger patients: 1.67; 95% CI: 1.38 to 2.03), treatment modification (n = 9304; aHR: 1.06; 95% CI: 0.97 to 1.17), and virologic failure (n = 7276; aHR: 0.81; 95% CI: 0.68 to 0.97).

## 4 Discussion

In our study of more than 26,000 HIV‐positive adults on ART in LAC, older age at time of ART initiation was associated with increased mortality, blunted CD4 recovery, and decreased risk of virologic failure. Given that the relative proportion of ART‐initiating patients over 50 years is increasing across the region, the results from this study provide important insight regarding the impact of ageing on ART outcomes for this growing population in the LAC region.

The clinical profile of patients in our cohort highlighted differences between older patients and their younger counterparts at the time of ART initiation. In our cohort, younger and older patients had similar CD4 counts and clinical AIDS status at the time of ART initiation; the same has been previously described in studies from resource‐limited settings from Sub‐Saharan Africa 31, 32. These findings contrast with earlier studies from high‐income countries, where older patients tended to have lower CD4 counts and more advanced disease compared to those who were younger 10. However, it is unclear whether these differences are due to temporal effects, given that the time periods for these studies are only partially overlapping. While our findings suggest that older patients are not being disproportionately marginalized from initiating care, the observed shorter length of time from HIV diagnosis to ART initiation in older patients may suggest a quicker engagement in care following diagnosis compared to younger persons.

The combination of higher mortality risk and blunted CD4 recovery despite less virologic failure among older patients in our cohort is not unique to the LAC region 31, 33, 34, 35. Studies from high‐income countries and resource‐limited settings have also shown higher rates of virologic suppression among older patients as a result of better treatment adherence compared to younger patients 12, 33, 36, 37, 38. Possible explanations for a higher mortality risk in older patients include multi‐morbidities, polypharmacy, chronic infection with viral pathogens (e.g. CMV), lower CD4/CD8 ratio, or other age‐related factors, as well as an increased risk of AIDS‐related complications given poor immunologic recovery despite ART 1, 9. A recent study by Edwards *et al*. highlighted the importance of ART initiation at higher CD4 counts in older adults to improve survival benefit 13. Considering the median CD4 count at ART initiation was <200 cells/μl for all adults in our cohort, there is a need for earlier diagnosis and ART initiation in the LAC region, especially for older patients.

In our study, there was no difference in ART modification by age group over time in the overall adjusted Cox regression analysis. Studies from high‐income regions have shown conflicting results regarding the association of older age and need for ART modification with some studies indicating a decreased risk of regimen switch and others showing an increased likelihood with older age 39, 40. Studies that have shown increased risk of ART modification for older adults have attributed the observation to higher rates of drug toxicities and adverse events 40. A recent study from Torres *et al*. in Brazil noted a trend where rates of drug toxicities and ART modification during the first year of therapy increased with age 41. Consistent with this, we also found in this study a trend towards a slightly increased risk of ART modification in older adults within the first three years of ART initiation. Conversely, older age was associated with a decreased risk of ART modification after the first three years in this study. While the reasons for ART modification were not available for our study, it is possible that drug toxicities and adverse events disproportionately contribute to early ART modification while virologic outcomes are greater determinants of later changes.

The availability and tolerability of ART regimens has substantially changed during the 20‐year span of this study. Our study sites are middle to low‐income countries and drug access is uneven in Latin America and the Caribbean. Although better and less toxic drug regimens exist, they were not available for free or were prescribed only for particular cases at our sites. For example, ART was provided for free by the Ministry of Health in Peru since the end of 2004, but treatment was very conservative and influenced by costs. Protease inhibitors (PIs) and newer drug classes were only prescribed under special circumstances. Between 2005 and 2010, there was a considerable boost in ART initiation at CCASAnet sites that corresponded to improved drug access, changes in guideline recommendations, and increased national campaigns. While rates of treatment modification varied over time, the increased risk in older adults relative to young patients remained consistent.

Lastly, our analyses of outcomes stratified by younger and older age groups revealed important findings regarding how patient characteristics at the time of ART initiation may influence treatment outcomes by age. Most strikingly was the observation that increasing age was associated with risk of mortality in both those age <50 years and those ≥50 years, underscoring the continuous effect of ageing and risk of mortality. Secondly, we found that the sex differences in ART modification and virologic failure observed in our primary analysis were mitigated and no longer statistically significant when analyses were restricted to those ≥50 years of age. It is possible that sex differences in healthcare‐seeking behaviour and family responsibilities, including pregnancy, that occur in early adulthood but are less pronounced in later years of life explain this difference. However, we also note that the relative frequency of men who had sex with men (MSM) was lower in the older age group. It is possible that the observed differences in association by sex between older and younger patients are due to unmeasured confounding. Lastly, increased risk of virologic failure associated with PI use (compared to NNRTI) was only observed in patients <50 years of age. For those over 50 years of age, PI use remained associated with increased likelihood of ART modification, underscoring questions about tolerability, side effects and the role of co‐morbidities rather than virologic failure as reasons for ART modification in older patients.

Age specific mortality rates of older adults included in the study were higher than age specific mortality rates reported by PAHO for the entire population. The difference is likely due to multiple factors such as immunosenescense and inflammation due HIV infection, ART toxicity, stigma and discrimination, higher risk of substance use, higher risk of malignancies and opportunistic diseases than HIV‐uninfected counterparts. We note, however, that the relative differences between mortality rates from our cohort and the general population decreased in older age groups. Given that death data sources differ by country and certain countries represented in CCASAnet (including Haiti) are not included in the reported PAHO regional estimates, comparisons of mortality between the HIV clinical cohort of CCASAnet and the general population should be interpreted with prudence.

A noteworthy limitation of our study is the limited availability of data regarding key indicators, including treatment adherence, reasons for ART modification, comorbidities and causes of death. A large percentage of patients (36%) were lost to follow‐up during this 20‐year observational study and have unknown outcomes. Lack of cause of death limits our ability to classify deaths as HIV‐related or age‐related. Given that mortality is expected to increase with age, we cannot be certain that associations between age and mortality in this study are related to HIV. Further study of the role of co‐morbidities (notably cardiovascular, renal, and liver disease), polypharmacy, and ART toxicity in older HIV‐positive adults in this region is needed to address these lingering questions and to guide therapy in this important patient population.

As with other studies using routinely collected clinical care data, key variables were missing for some patients in our study. The lack of VL data for GHESKIO‐Haiti required the exclusion of the entire site from analyses involving virologic failure outcomes; an additional 4174 patients (16%) were excluded from other sites. Furthermore, over 60% of the cohort was missing at least one covariate. Reasons for data being missing for specific covariates vary. Laboratory measurements such as CD4 and VL were collected less frequently at some sites because of costs in the earlier years of ART and due to changing guidelines for virologically suppressed patients in later years; HIV diagnosis date was sometimes unknown by clinics; probable route of HIV transmission was sometimes not recorded or categorized as unknown by patient self‐report; clinical AIDS status at baseline was not always available from medical records. Although results were similar between complete case and multiply imputed analyses, these high levels of missingness merit prudence when interpreting study findings.

## 5 Conclusions

Our study highlights the higher risk of death among older patients on ART in LAC, while lower risk of virologic failure and similar risk of ART modification. This study represents, to our knowledge, the first analysis of ART outcomes focused on older HIV populations in Latin America and the Caribbean. The generalizability of our findings is strengthened by the number of patients contributing data from multiple, diverse sites across the region. Given the complexity of issues related to ageing with HIV, a greater understanding is needed in order to properly respond to this shifting epidemic.

## Competing interests

The authors declare that they have no competing interests.

## Authors’ contributions

GC, MG, KJ, PC, BG, MW, JWP, DP, JS, EG, CM and BS conceived and designed the study. GC, MG, KJ and EG performed data abstraction. KJ contributed to data management. GC, MG, JC, BS and EG analysed the data. GC, MG and JC wrote the draft manuscript. GC, MG, JC, KJ, PC, BG, MW, JWP, DP, JS, EG, CM and BS contributed to the critical appraisal of the manuscript. GC, MG, JC, KJ, PC, BG, MW, JWP, DP, JS, EG, CM and BS approved the final version of the manuscript.

## Supporting information


**Figure S1.** Crude cumulative incidence of regimen modification by age group and timing of ART initiation.
**Table S1.** Comparison of age‐specific mortality rates (per 100,000 person years) between CCASAnet patients and the general population using 2014 data from the Pan‐American Health Organization
**Table S2.** Comparison of age‐specific mortality rates (per 100,000 person years) by country between CCASAnet patients and the general population using data from the Pan‐American Health Organization
**Table S3.** Comparison of all‐cause mortality, treatment modification and virologic failure between older and younger patients
**Table S4.** Comparison of hazard ratios for all‐cause mortality, treatment modification and virologic failure among patients ≥50 years old
**Table S5.** Comparison of hazard ratios for all‐cause mortality, treatment modification and virologic failure among patients <50 years old
**Table S6.** A comparison of imputed values (from a single replication) and observed values for all variables with missing observations
**Table S7.** Comparison of all‐cause mortality (n = 9304), treatment modification (n = 9304) and virologic failure (n = 7276) between older and younger patients USING ONLY COMPLETE CASES DATA
