# Supplementary material for: Virologic failure and mortality in older ART initiators in a multisite Latin American and Caribbean Cohort
Source: J Int AIDS Soc. 2018 Mar 22;21(3):e25088. doi: 10.1002/jia2.25088 (PMC5864576; doi:10.1002/jia2.25088)
Supplement: Supplementary file 1 — Figure S1. Crude cumulative incidence of regimen modification by age group and timing of ART initiation. Table S1. Comparison of age‐specific mortality rates (per 100,000 person years) between CCASAnet patients and the general population using 2014 data from the Pan‐American Health Organization Table S2. Comparison of age‐specific mortality rates (per 100,000 person years) by country between CCASAnet patients and the general population using data from the Pan‐American Health Organization Table S3. Comparison of all‐cause mortality, treatment modification and virologic failure between older and younger patients Table S4. Comparison of hazard ratios for all‐cause mortality, treatment modification and virologic failure among patients ≥50 years old Table S5. Comparison of hazard ratios for all‐cause mortality, treatment modification and virologic failure among patients <50 years old Table S6. A comparison of imputed values (from a single replication) and observed values for all variables with missing observations Table S7. Comparison of all‐cause mortality (n = 9304), treatment modification (n = 9304) and virologic failure (n = 7276) between older and younger patients USING ONLY COMPLETE CASES DATA [file JIA2-21-e25088-s001.docx]

**Supplemental Figure 1.** Crude cumulative incidence of regimen modification by age group and timing of ART initiation.

**
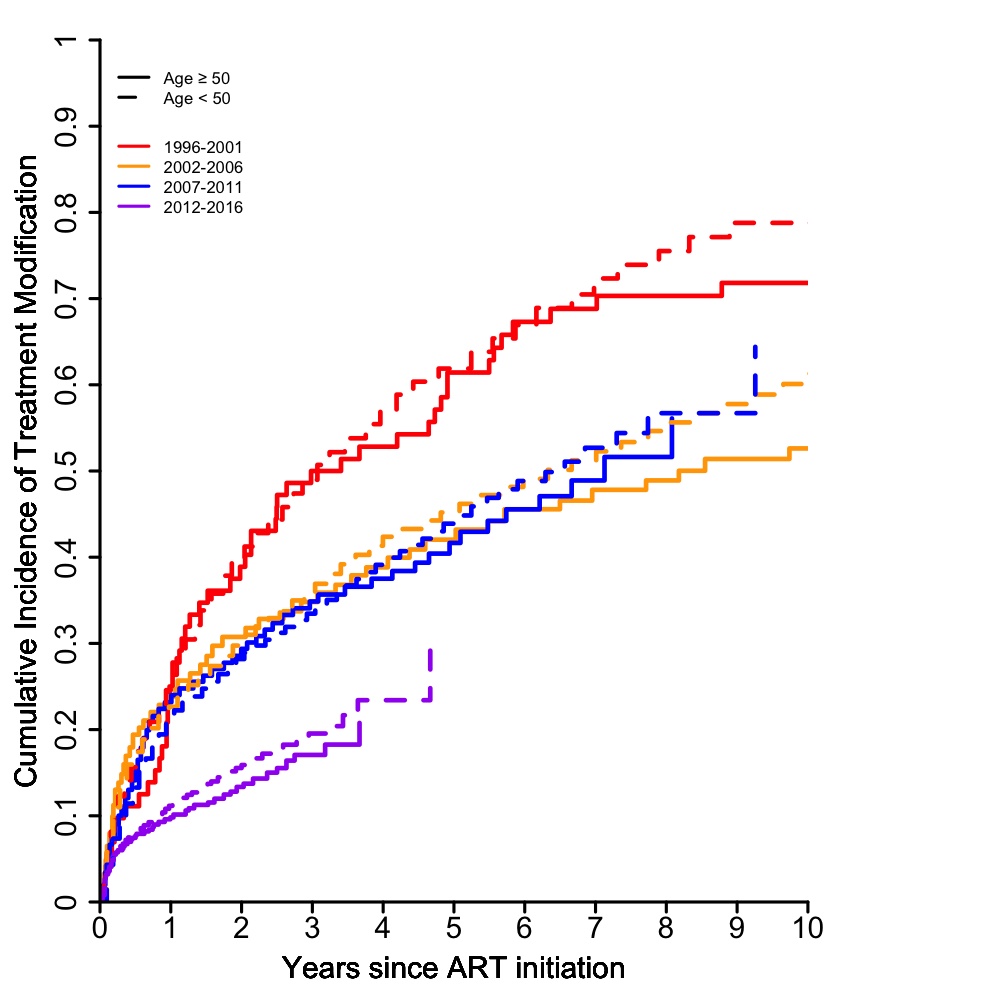
**

**Supplemental Table 1: Comparison of age-specific mortality rates (per 100,000 person years) between CCASAnet patients and the general population using 2014 data from the Pan-American Health Organization.** Regional estimates for South America and Central America may contain data for countries not represented in CCASAnet.

|  | CCASAnet  (7 countries) | PAHO – South America  (2014 – 8 countries) | PAHO – Central America  (2014 – 4 countries) |
| --- | --- | --- | --- |
| 20-24 | 1449 (1082 - 1833) | 142 | 172 |
| 25-29 | 1933 (1704 - 2197) |  |  |
| 30-34 | 1763 (1585 - 1967) |  |  |
| 35-39 | 1779 (1614 - 1958) | 213 | 253 |
| 40-44 | 1856 (1675 - 2044) |  |  |
| 45-49 | 2061 (1846 - 2278) | 424 | 452 |
| 50-54 | 2321 (2054 - 2623) |  |  |
| 55-59 | 2500 (2111 - 2888) | 937 | 943 |
| 60-64 | 2999 (2433 - 3606) |  |  |
| 65-69 | 2627 (1867 - 3425) | 2149 | 1875 |
| 70-74 | 5225 (3616 - 6969) |  |  |
| 75+ | 6201 (3507 - 9595) | 7632 | 6996 |

|  | **Argentina** | | **Brazil** | | **Chile** | | **Haiti** | | **Honduras** | | **Mexico** | | **Peru** | |
| --- | --- | --- | --- | --- | --- | --- | --- | --- | --- | --- | --- | --- | --- | --- |
|  | CCASAnet | PAHO | CCASAnet | PAHO | CCASAnet | PAHO | CCASAnet | PAHO | CCASAnet | PAHO | CCASAnet | PAHO | CCASAnet | PAHO |
|  |  | (2014) |  | (2014) |  | (2014) |  | (2004) |  | (2013) |  | (2014) |  | (2014) |
| Age Group |  |  |  |  |  |  |  |  |  |  |  |  |  |  |
| **20-34** | 518 | 106 | 1646 | 168 | 1062 | 76 | 2537 | 16 | 2471 | 25 | 627 | 123 | 1709 | 71 |
|  | (264 - 820) |  | (1305 - 2023) |  | (730 - 1370) |  | (2243 - 2836) |  | (1747 - 3361) |  | (311 - 983) |  | (1382 - 2052) |  |
|  |  |  |  |  |  |  |  |  |  |  |  |  |  |  |
| **35-44** | 713 | 172 | 1835 | 244 | 1215 | 135 | 2358 | 25 | 2432 | 48 | 909 | 214 | 1758 | 119 |
|  | (469 - 962) |  | (1518 - 2226) |  | (936 - 1500) |  | (2127 - 2591) |  | (1824 - 3112) |  | (551 - 1338) |  | (1415 - 2130) |  |
|  |  |  |  |  |  |  |  |  |  |  |  |  |  |  |
| **45-54** | 792 | 405 | 2348 | 471 | 1535 | 301 | 2543 | 40 | 2619 | 92 | 908 | 458 | 2391 | 230 |
|  | (480 - 1146) |  | (1885 - 2823) |  | (1132 - 1990) |  | (2253 - 2857) |  | (1824 - 3602) |  | (382 - 1520) |  | (1853 - 2949) |  |
|  |  |  |  |  |  |  |  |  |  |  |  |  |  |  |
| **55-64** | 1036 | 1029 | 3435 | 1013 | 1613 | 698 | 3448 | 47 | 1583 | 184 | 1553 | 1014 | 2078 | 495 |
|  | (468 - 1705) |  | (2579 - 4475) |  | (999 - 2329) |  | (2911 - 4024) |  | (464 - 2990) |  | (485 - 2975) |  | (1332 - 3062) |  |

**Supplemental Table 2: Comparison of age-specific mortality rates (per 100,000 person years) by country between CCASAnet patients and the general population using data from the Pan-American Health Organization.**

**Supplemental Table 3. Comparison of all-cause mortality, treatment modification, and virologic failure between older and younger patients.**

|  | | **Death**  **Hazard Ratio** | **ART Modification**  **Hazard Ratio** | **Virologic Failure**  **Hazard Ratio** |
| --- | --- | --- | --- | --- |
| Age group | |  |  |  |
|  | *<* 50 years | Ref | Ref | Ref |
|  | ≥ 50 years | 1.64 (1.48 - 1.83) | 1.00 (0.94 - 1.06) | 0.73 (0.63 - 0.84) |
| Sex | |  |  |  |
|  | Female | Ref | Ref | Ref |
|  | Male | 1.10 (1.00 - 1.20) | 0.82 (0.78 - 0.85) | 0.76 (0.69 - 0.84) |
| Clinical stage, baseline | |  |  |  |
|  | Not AIDS | Ref | Ref | Ref |
|  | AIDS | 1.64 (1.49 - 1.81) | 1.17 (1.11 - 1.23) | 1*.*05 (0*.*91 - 1*.*21) |
| Nadir CD4 count, cells/µL | |  |  |  |
|  | 50 | 2*.*54 (2*.*20 - 2*.*93) | 1*.*55 (1*.*45 - 1*.*65) | 1*.*31 (1*.*15 - 1*.*49) |
|  | 100 | 2*.*00 (1*.*74 - 2*.*31) | 1*.*43 (1*.*34 - 1*.*52) | 1*.*26 (1*.*11 - 1*.*43) |
|  | 200 | 1*.*33 (1*.*23 - 1*.*43) | 1*.*22 (1*.*18 - 1*.*26) | 1*.*06 (0*.*99 - 1*.*13) |
|  | 350 | Ref | Ref | Ref |
| ART initiation year | |  |  |  |
|  | 2000 | 1*.*10 (0*.*90 - 1*.*34) | 0*.*69 (0*.*63 - 0*.*75) | 2*.*24 (1*.*92 - 2*.*62) |
|  | 2004 | 1*.*13 (1*.*06 - 1*.*22) | 0*.*89 (0*.*86 - 0*.*92) | 1*.*37 (1*.*28 - 1*.*48) |
|  | 2008 | Ref | Ref | Ref |
|  | 2012 | 0*.*70 (0*.*63 - 0*.*78) | 0*.*76 (0*.*73 - 0*.*81) | 0*.*99 (0*.*90 - 1*.*09) |
|  | 2016 | 0*.*64 (0*.*46 - 0*.*88) | 0*.*38 (0*.*33 - 0*.*45) | 1*.*18 (0*.*84 - 1*.*65) |
| ART regimen class | |  |  |  |
|  | NNRTI | Ref | Ref | Ref |
|  | PI | 1*.*13 (0*.*97 - 1*.*32) | 1*.*24 (1*.*16 - 1*.*32) | 1*.*27 (1*.*12 - 1*.*43) |
|  | Other | 1*.*04 (0*.*85 - 1*.*27) | 2*.*37 (2*.*17 - 2*.*58) | 1*.*57 (1*.*34 - 1*.*84) |
| History of IDU | |  |  |  |
|  | No | Ref | Ref | Ref |
|  | Yes | 1*.*46 (0*.*95 - 2*.*24) | 1*.*08 (0*.*89 - 1*.*30) | 1*.*21 (0*.*87 - 1*.*68) |
| Time from HIV Diagnosis to ART, years | |  |  |  |
|  | 0 | Ref | Ref | Ref |
|  | 6 months | 1*.*01 (0*.*97 - 1*.*04) | 1*.*00 (0*.*98 - 1*.*02) | 0*.*93 (0*.*90 - 0*.*97) |
|  | 1 year | 1*.*01 (0*.*95 - 1*.*08) | 1*.*00 (0*.*97 - 1*.*03) | 0*.*88 (0*.*82 - 0*.*94) |
|  | 3 years | 1*.*00 (0*.*89 - 1*.*13) | 1*.*00 (0*.*94 - 1*.*06) | 0*.*76 (0*.*67 - 0*.*86) |

**Supplemental Table 4. Comparison of hazard ratios for all-cause mortality, treatment modification, and virologic failure among patients ≥ 50 years old.**

|  |  | **Death** | **ART Modification** | **Virologic Failure** |
| --- | --- | --- | --- | --- |
| Age, years |  |  |  |  |
|  | 50 | Ref | Ref | Ref |
|  | 60 | 1*.*48 (1*.*10 - 1*.*98) | 1*.*05 (0*.*87 - 1*.*28) | 0*.*91 (0*.*59 - 1*.*40) |
|  | 70 | 2*.*19 (1*.*59 - 3*.*02) | 1*.*09 (0*.*85 - 1*.*40) | 0*.*91 (0*.*50 - 1*.*68) |
|  | 80 | 3*.*41 (2*.*06 - 5*.*65) | 1*.*13 (0*.*71 - 1*.*81) | 0*.*98 (0*.*31 - 3*.*12) |
| Sex |  |  |  |  |
|  | Female | Ref | Ref | Ref |
|  | Male | 1*.*12 (0*.*92 - 1*.*37) | 0*.*98 (0*.*85 - 1*.*13) | 0*.*86 (0*.*63 - 1*.*17) |
| Clinical stage at baseline |  |  |  |  |
|  | Not AIDS | Ref | Ref | Ref |
|  | AIDS | 1*.*52 (1*.*20 - 1*.*92) | 1*.*15 (0*.*96 - 1*.*38) | 1*.*41 (0*.*95 - 2*.*08) |
| Nadir CD4 count, cells/µL |  |  |  |  |
|  | 50 | 2*.*00 (1*.*47 - 2*.*72) | 1*.*67 (1*.*35 - 2*.*05) | 1*.*42 (0*.*91 - 2*.*20) |
|  | 100 | 1*.*77 (1*.*30 - 2*.*42) | 1*.*48 (1*.*20 - 1*.*82) | 1*.*34 (0*.*89 - 2*.*01) |
|  | 200 | 1*.*35 (1*.*14 - 1*.*60) | 1*.*23 (1*.*10 - 1*.*37) | 0*.*99 (0*.*80 - 1*.*24) |
|  | 350 | Ref | Ref | Ref |
| ART initiation year |  |  |  |  |
|  | 2000 | 0*.*93 (0*.*57 - 1*.*51) | 0*.*56 (0*.*40 - 0*.*79) | 1*.*69 (0*.*97 - 2*.*94) |
|  | 2004 | 1*.*08 (0*.*90 - 1*.*29) | 0*.*81 (0*.*71 - 0*.*92) | 1*.*28 (1*.*01 - 1*.*63) |
|  | 2008 | Ref | Ref | Ref |
|  | 2012 | 0*.*60 (0*.*47 - 0*.*78) | 0*.*71 (0*.*60 - 0*.*84) | 0*.*99 (0*.*73 - 1*.*34) |
|  | 2016 | 0*.*56 (0*.*28 - 1*.*15) | 0*.*28 (0*.*18 - 0*.*45) | 1*.*39 (0*.*47 - 4*.*09) |
| ART regimen class |  |  |  |  |
|  | NNRTI | Ref | Ref | Ref |
|  | PI | 1*.*00 (0*.*70 - 1*.*43) | 1*.*34 (1*.*08 - 1*.*65) | 0*.*98 (0*.*63 - 1*.*53) |
|  | Other | 1*.*34 (0*.*86 - 2*.*10) | 2*.*35 (1*.*77 - 3*.*13) | 1*.*64 (1*.*03 - 2*.*61) |
| History of IDU |  |  |  |  |
|  | No | NA | NA | NA |
|  | Yes | NA | NA | NA |
| Time from HIV Diagnosis to ART, years |  |  |  |  |
|  | 0 | Ref | Ref | Ref |
|  | 6 months | 0*.*97 (0*.*90 - 1*.*06) | 1*.*03 (0*.*97 - 1*.*09) | 0*.*90 (0*.*79 - 1*.*01) |
|  | 1 year | 0*.*95 (0*.*82 - 1*.*11) | 1*.*05 (0*.*94 - 1*.*17) | 0*.*81 (0*.*65 - 1*.*01) |
|  | 3 years | 0*.*90 (0*.*67 - 1*.*21) | 1*.*10 (0*.*90 - 1*.*35) | 0*.*64 (0*.*42 - 0*.*99) |

**Supplemental Table 5. Comparison of hazard ratios for all-cause mortality, treatment modification, and virologic failure among patients < 50 years old.**

|  |  | **Death** | **ART Modification** | **Virologic Failure** |
| --- | --- | --- | --- | --- |
| Age, years |  |  |  |  |
|  | 50 | Ref | Ref | Ref |
|  | 40 | 0*.*76 (0*.*62 - 0*.*92) | 0*.*87 (0*.*77 - 0*.*99) | 1*.*10 (0*.*87 - 1*.*38) |
|  | 30 | 0*.*70 (0*.*58 - 0*.*83) | 0*.*94 (0*.*85 - 1*.*05) | 1*.*33 (1*.*08 - 1*.*63) |
|  | 20 | 0*.*81 (0*.*62 - 1*.*05) | 0*.*95 (0*.*82 - 1*.*11) | 2*.*04 (1*.*55 - 2*.*67) |
|  |  |  |  |  |
| Sex | Female | Ref | Ref | Ref |
|  | Male | 1.09 (0.99 - 1.20) | 0.77 (0.73 - 0.81) | 0.75 (0.68 - 0.83) |
|  |  |  |  |  |
| Clinical stage at baseline | Not AIDS | Ref | Ref | Ref |
|  | AIDS | 1*.*66 (1*.*49 - 1*.*85) | 1*.*25 (1*.*17 - 1*.*34) | 1*.*03 (0*.*88 - 1*.*20) |
|  |  |  |  |  |
| Nadir CD4 count, cells/µL | 50 | 2*.*69 (2*.*29 - 3*.*17) | 1*.*58 (1*.*46 - 1*.*71) | 1*.*35 (1*.*18 - 1*.*55) |
|  | 100 | 2*.*07 (1*.*77 - 2*.*43) | 1*.*46 (1*.*35 - 1*.*58) | 1*.*30 (1*.*13 - 1*.*48) |
|  | 200 | 1*.*32 (1*.*21 - 1*.*45) | 1*.*26 (1*.*20 - 1*.*31) | 1*.*09 (1*.*01 - 1*.*17) |
|  | 350 | Ref | Ref | Ref |
|  |  |  |  |  |
| ART initiation year | 2000 | 1*.*18 (0*.*95 - 1*.*46) | 0*.*76 (0*.*68 - 0*.*85) | 2*.*32 (1*.*97 - 2*.*73) |
|  | 2004 | 1*.*15 (1*.*07 - 1*.*25) | 0*.*93 (0*.*89 - 0*.*97) | 1*.*39 (1*.*29 - 1*.*50) |
|  | 2008 | Ref | Ref | Ref |
|  | 2012 | 0*.*73 (0*.*64 - 0*.*83) | 0*.*77 (0*.*72 - 0*.*82) | 0*.*97 (0*.*88 - 1*.*07) |
|  | 2016 | 0*.*66 (0*.*46 - 0*.*94) | 0*.*44 (0*.*37 - 0*.*52) | 1*.*11 (0*.*77 - 1*.*58) |
|  |  |  |  |  |
| ART regimen class | NNRTI | Ref | Ref | Ref |
|  | PI | 1*.*14 (0*.*97 - 1*.*35) | 1*.*20 (1*.*11 - 1*.*30) | 1*.*28 (1*.*13 - 1*.*46) |
|  | Other | 0*.*98 (0*.*78 - 1*.*22) | 2*.*14 (1*.*93 - 2*.*39) | 1*.*55 (1*.*31 - 1*.*84) |
|  |  |  |  |  |
| History of IDU | No | NA | NA | NA |
|  | Yes | NA | NA | NA |
|  |  |  |  |  |
| Time from HIV Diagnosis to ART, years | 0 | Ref | Ref | Ref |
|  | 6 months | 1*.*01 (0*.*97 - 1*.*05) | 1*.*00 (0*.*98 - 1*.*02) | 0*.*94 (0*.*90 - 0*.*97) |
|  | 1 year | 1*.*02 (0*.*94 - 1*.*09) | 0*.*99 (0*.*96 - 1*.*04) | 0*.*88 (0*.*83 - 0*.*95) |
|  | 3 years | 1*.*01 (0*.*88 - 1*.*15) | 0*.*99 (0*.*92 - 1*.*07) | 0*.*77 (0*.*67 - 0*.*87) |

**Supplemental Table 6. A comparison of imputed values (from a single replication) and observed values for all variables with missing observations.** Since multiple imputation procedure assumes missingness at random, no statistical test for differences between imputed and observed values is performed.

|  | | **Imputed values** | **Observed values** |
| --- | --- | --- | --- |
| Probable route of transmission | |  |  |
|  | IVDU | 123(1%) | 202(2%) |
|  | No IVDU | 13484 (99%) | 12502 (98%) |
| Clinical stage, baseline | |  |  |
|  | AIDS | 798(16%) | 5041(24%) |
|  | Not AIDS | 4104(84%) | 16368(76%) |
| Nadir CD4 count, cells/µL | | n= 3085; 186 (86 - 312) | n = 23226; 182 (72 - 295) |
| Time from HIV diagnosis to ART initiation, days | | n = 299; 545*.*1 (-163*.*9 - 1222) | n = 26012; 146 (34 - 862) |

**Supplemental Table 7. Comparison of all-cause mortality (n=9304), treatment modification (n=9304), and virologic failure (n=7276) between older and younger patients USING ONLY COMPLETE CASES DATA.**

|  | | **Death**  **Hazard Ratio** | **ART Modification**  **Hazard Ratio** | **Virologic Failure**  **Hazard Ratio** |
| --- | --- | --- | --- | --- |
| Age group | |  |  |  |
|  | *<* 50 years | Ref | Ref | Ref |
|  | ≥ 50 years | 1.67 (1.38 - 2.03) | 1.06 (0.97 - 1.17) | 0.81 (0.68 - 0.97) |
| Sex | |  |  |  |
|  | Female | Ref | Ref | Ref |
|  | Male | 1*.*10 (0*.*92 - 1*.*31) | 0*.*76 (0*.*71 - 0*.*82) | 0*.*76 (0*.*68 - 0*.*86) |
| Clinical AIDS, baseline | |  |  |  |
|  | No | Ref | Ref | Ref |
|  | Yes | 1*.*55 (1*.*31 - 1*.*84) | 1*.*06 (0*.*99 - 1*.*14) | 1*.*06 (0*.*93 - 1*.*21) |
| Nadir CD4 count, cells/µL | |  |  |  |
|  | 50 | 2*.*38 (1*.*88 - 3*.*01) | 1*.*54 (1*.*41 - 1*.*69) | 1.39 (1.19 - 1.63) |
|  | 100 | 1*.*81 (1*.*45 - 2*.*28) | 1*.*39 (1*.*28 - 1*.*52) | 1*.*32 (1.13 - 1*.*53) |
|  | 200 | 1.24 (1.07 - 1.44) | 1.16 (1.11 – 1.22) | 1*.*09 (1*.*00 - 1*.*18) |
|  | 350 | Ref | Ref | Ref |
| ART initiation year | |  |  |  |
|  | 2000 | 1*.*24 (0*.*90 - 1*.*72) | 0*.*87 (0*.*77 - 1*.*00) | 2*.*60 (2*.*12 - 3*.*18) |
|  | 2004 | 1*.*07 (0*.*95 - 1*.*21) | 0*.*95 (0*.*90 - 0*.*99) | 1*.*45 (1*.*34 - 1*.*57) |
|  | 2008 | Ref | Ref | Ref |
|  | 2012 | 0*.*97 (0*.*80 - 1*.*17) | 0*.*99 (0*.*91 - 1*.*06) | 1*.*02 (0*.*90 - 1*.*16) |
|  | 2016 | 0*.*62 (0*.*39 - 0*.*99) | 0*.*88 (0*.*73 - 1*.*06) | 1*.*10 (0*.*70 - 1*.*73) |
| ART regimen class | |  |  |  |
|  | NNRTI | Ref | Ref | Ref |
|  | PI | 1*.*28 (1*.*03 - 1*.*60) | 1*.*31 (1*.*20 - 1*.*43) | 1*.*23 (1*.*06 - 1*.*43) |
|  | Other | 0*.*96 (0*.*68 - 1*.*36) | 1*.*98 (1*.*75 - 2*.*25) | 1*.*61 (1*.*32 - 1*.*97) |
| History of IDU | |  |  |  |
|  | No | Ref | Ref | Ref |
|  | Yes | 1.80 (1.06 - 3.05) | 1.04 (0.83 - 1.31) | 1.27 (0.85 - 1.89) |
| Time from HIV Diagnosis to ART, years | |  |  |  |
|  | 0 | Ref | Ref | Ref |
|  | 6 months | 0*.*99 (0*.*92 - 1*.*07) | 1*.*00 (0*.*97 - 1*.*03) | 0*.*93 (0*.*88 - 0*.*98) |
|  | 1 year | 0*.*98 (0*.*85 - 1*.*12) | 1*.*00 (0*.*95 - 1*.*06) | 0*.*87 (0*.*79 - 0*.*96) |
|  | 3 years | 0*.*93 (0*.*73 - 1*.*17) | 0*.*99 (0*.*90 - 1*.*09) | 0*.*78 (0*.*66 - 0*.*93) |
